# Supplementary material for: Causal Relationship between Aging and Anorexia Nervosa: A White-Matter-Microstructure-Mediated Mendelian Randomization Analysis
Source: Biomedicines. 2024 Aug 16;12(8):1874. doi: 10.3390/biomedicines12081874 (PMC11351342; doi:10.3390/biomedicines12081874)
Supplement: Supplementary file 1 [file biomedicines-12-01874-s001.zip › Supplementary Figure/Figure S7/Single SNP plot of MR analysis for ubm-b-1894 to AN.pdf]

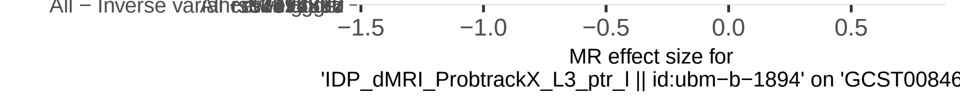
All − Inverse variance weighted
−1.5
−1.0
−0.5
0.0
0.5
MR effect size for
'IDP_dMRI_ProbtrackX_L3_ptr_l || id:ubm−b−1894' on 'GCST00846
